# A preoperative scoring system to predict the probability of laparoendoscopic single-site extracorporeal cystectomy in patients with benign ovarian cysts

**DOI:** 10.3389/fsurg.2022.991450

**Published:** 2022-10-26

**Authors:** Wenwei Tan, Yuan Deng, Li Deng, Shuai Tang, Yuanyang Yao, Huanyu Wei, Kuiyan Zhong, Yanzhou Wang

**Affiliations:** Department of Obstetrics and Gynecology, First Affiliated Hospital, Army Medical University, Chongqing, China

**Keywords:** laparoendoscopic single-site, extracorporeal, cystectomy, benign ovarian cysts, preoperative scoring system

## Abstract

**Objective:**

To develop a preoperative scoring system (PSS) to predict whether laparoendoscopic single-site extracorporeal (LESS-E) cystectomy can be performed in patients with benign ovarian cysts.

**Method:**

We reviewed data on patients who underwent LESS cystectomy between August 2016 and October 2019 at the first Affiliated Hospital, Army Medical University. The independent predictors of LESS-E cystectomy in patients with benign ovarian cysts were identified using multivariate logistic regression analyses. A nomogram for predicting LESS-E cystectomy in patients with benign ovarian cysts was developed, and to simplify the score, we establish a preoperative scoring system to guide the choice of surgical approach in patients with highly probable benign ovarian cysts.

**Results:**

Our analysis showed that age, BMI, height and the diameter of ovarian cysts were independent predictors of LESS-E cystectomy. A nomogram was developed based on these four factors, which had a concordance index of 0.838 and *R*^2 ^= 0.415. To simplify the score, the predicted indicators in the regression model were scored by dividing the beta coefficient by the absolute value of the minimum beta coefficient, and the sum of each predictor score established a PSS. In the total set, the selected cutoff value according to the maximum point of the Youden index was 8, and a preoperative score ≥ 8 identified patients undergoing LESS-E cystectomy with a positive predictive value of 67.4% and a negative predictive value of 88.6%.

**Conclusion:**

A PSS to predict the chances of LESS-E cystectomy was established. This system could be helpful for selecting the appropriate surgical strategy for patients with benign ovarian cysts.

## Introduction

Ovarian cysts are one of the most common diseases in gynecology and usually occur at fertile ages (1). The laparoscopy procedure is regarded as the gold standard to cure benign ovarian cysts because of the less trauma, shorter hospital stay and faster recovery compared with laparotomy (2). Laparoendoscopic single-site (LESS) surgery has several advantages over conventional laparoscopic surgery (CLS), including decreased postoperative pain and improved cosmesis (3–7). The main disadvantages of LESS surgery are the technical challenges due to the limited space, and some doctors have difficulty mastering surgical tricks (8, 9), which may result in a prolonged operative time (10–12).

Meanwhile, some previous studies showed the superiority of a laparoendoscopic single-site extracorporeal (LESS-E) technique that combines the virtue of laparoendoscopic and laparotomy (13–16). The surgeons do the operation through the process of an open surgery, who get convenient and direct cystectomy, and the patients suffer less pain and recover faster, who get the advantage of minimally invasive surgery. To our knowledge, laparoscopy combined with the extracorporeal approach for ovarian cystectomy was first introduced in 2004 (17). Subsequently, some similar techniques were reported in the treatment of large ovarian cysts that could maximally preserve the normal ovarian issue while achieving cosmetic purposes (13, 17–22). The feasibility and safety of the laparoscopic-assisted extracorporeal approach were confirmed in these studies.

According to our retrospective, propensity score matched study (23), we collected the clinical data of 105 patients who underwent LESS-E cystectomy and 138 patients who conducted LESS-I cystectomy, comparing the perioperative results of the two groups after propensity score matching, it turned out that laparoendoscopic single-site extracorporeal (LESS-E) cystectomy had a shorter operation time and lower leakage rates than laparoendoscopic single-site intracorporeal (LESS-I) cystectomy in selected patients. Therefore, it is worth offering LESS-E cystectomy to patients with highly probable benign ovarian cysts based on pre-operative evaluation.

However, only 43% of patients in our previous study underwent LESS-E cystectomy (23). Here is the question: which patients are most suitable for LESS-E cystectomy? The purpose of this study was to establish a preoperative scoring system (PSS) to predict the probability of performing laparoendoscopic single-site extracorporeal cystectomy in patients with benign ovarian cysts as an evidence-based tool to guide patient classification and clinical treatment options. For patients who are unsuitable for LESS-E cystectomy, conventional laparoscopy, LESS-I surgery or reduced port approach can be performed according to the patient's willingness and the assessment of surgeons.

## Materials and methods

The study was approved by the Ethics Committee of First Affiliated Hospital, Army Medical University (Approval No: KY202024). Informed consent was waived because retrospective deidentified data were used. During LESS-E cystectomy, we performed adhesiolysis intracorporeally with single-port laparoscopy, the detachable port cap in the LESS-E group was then removed to start the extracorporeal process. After identifying the mass, a purse-string suture was used at the corners of the cystic surface, which was exposed with a wound retractor. The cyst was then punctured through the suture, and a suction tip was used to rapidly aspirate its contents. A Kelly clamp was used to hold the puncture site on the cyst, and pieces of surgical gauze were used to cover the internal edge of the wound retractor to prevent the spillage of cystic content. Upon exteriorization, the ovarian cyst was encircled with wet wide surgical gauze to avoid tissue dryness and extracorporeal spillage throughout the surgery. Next, common surgical instruments were used to completely separate the cystic capsule from the normal tissue as in open surgery, and the remaining ovarian tissue is remodeled with common surgical instruments (23).

**Figure 1:**
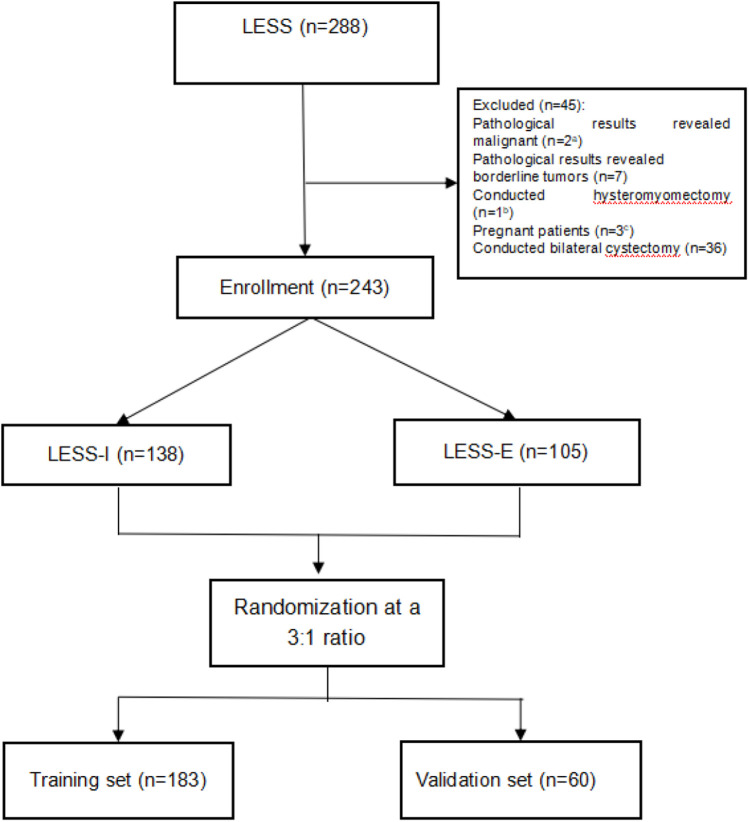
Flowchart of the training set and validation set. a, 1 of the 2 associated with bilateral cystectomy; b, associated with bilateral cystectomy; c, 2 of the 3 associated with bilateral cystectomy.

The clinical characteristics of patients who underwent LESS ovarian cystectomy in our hospital from August 1, 2016, to October 29, 2019, were retrospectively reviewed. The inclusion criteria were patients who successfully underwent LESS-E or LESS-I cystectomy. The exclusion criteria were as follows: (1) pathological results revealing malignant or borderline tumors; (2) hysteromyomectomy; (3) pregnancy; (4) bilateral cystectomy. Eventually, 243 patients with benign ovarian cysts who met the criteria were enrolled, including 105 patients who underwent LESS-E cystectomy and 138 patients who underwent LESS-I cystectomy. Patients were randomly allocated into the training set and validation set based on a 3:1 ratio, with 183 patients included in the training set and 60 patients included in the validation set (Figure 1).

All preoperative indicators, including age, height, body mass index (BMI), gravidity, parity, cesarean section, history of abdominal surgery, relapse of cyst, preoperative hemoglobin, preoperative CA 125, other tumor markers, location of the cyst, largest diameter of the cyst, deduced cyst pathological type pre-operatively and ultrasound findings, were collected. The deduced pathological types consisted of mature cystic teratoma, endometrioma, mucinous/serous cystadenoma, mesosalpinx cyst, parovarian cyst, and simple cyst. Ultrasound findings revealed cystic, mixed cyst-solid, dense spot echo and multilocular features.

## Statistical analysis

### Development and validation of the nomogram

According to the receiver operating characteristic (ROC) curve of preoperative indicators such as age, height, BMI and the largest diameter of the cyst, the maximum value of the Youden index was selected as the boundary value to convert the continuous variables into categorical variables. A multivariate logistic regression analysis of preoperative indicators was performed with the training set, and a nomogram combining independent predictors and clinical factors was developed. The nomogram was internally and externally validated with the training set and validation set, respectively, using the bootstrapping technique (1,000 repetitions). Decision curve analysis (DCA) was employed to assess the clinical value of the nomogram by calculating the net benefit for patients at each threshold probability.

### Development of the PSS

To simplify the score, the predicted indicators in the regression model were scored by dividing the beta coefficient by the absolute value of the minimum beta coefficient (24). A score of 0 was used for the variables for which a woman had a reverse result. The sum of each predictor score was used to establish the PSS. The ROC curve of the PSS was generated with the training set and the validation set, and the area under the receiver-operating characteristic curve (AUC) was used to measure the model's discriminatory power. It is generally accepted that an AUC of 1.0 demonstrates perfect accuracy, values > 0.8 represent good discrimination, an AUC of 0.7–0.8 shows satisfactory discrimination, and an AUC of 0.5 indicates no relationship.

### Optimal threshold of the PSS

The optimal threshold (cutoff points) of the PSS in terms of specificity, sensitivity, positive predictive values (PPVs), and negative predictive values (NPVs) was evaluated by the Youden index according to the ROC curve of the total set (25).

## Other statistical analyses

Statistical analysis was performed using SPSS 22.0 and R statistical software 4.0.3. Consecutive variables that conformed to a normal distribution are expressed as the mean ± standard deviation (SD), and t tests were used for comparison of the training set and validation set. Categorical variables are expressed as frequencies (percentages) and were analyzed using the chi-square test or Fisher's exact test. Missing data in the predictors were multiply imputed with chained equations (MICE) before statistical analysis to avoid the expurgation of potentially essential data and the introduction of selection bias if only unabridged cases were applied to the analysis (26). A *P* value less than 0.05 was considered to indicate statistical significance.

## Results

### Characteristics of the study population

The preoperative characteristics of 243 patients who met the inclusion criteria are summarized in Table 1. Patients were randomly assigned to the training set (*n* = 183) or validation set (*n* = 60). The mean age in the training set and validation set was 28 ± 8.01 years and 27.3 ± 7.98 years, respectively (*P* = 0.579). The mean (SD) largest diameter of ovarian cysts was 8.74 (3.85) cm in the training set and 8.12 (3.41) cm in the validation set (*P* = 0.270). BMI values were similar between the groups (*P* = 0.926). There was no significant difference between the two groups, except for the inferred pathological type before surgery. It is worth mentioning that whether conducted extracorporeal approach or not was determined by surgeon during operation and no conversion of surgical approach occurred during the operation. The comparison between the LESS-E group and the LESS-I group is shown in Supplementary Table S1. Forty-three percent of patients in our study underwent LESS-E cystectomy.

**Table 1:** Patient characteristics.

	Training set (*N* = 183)	Validation set (*N* = 60)	Statistical parameters	*P*
Age (years)	28.0 ± 8.01	27.3 ± 7.98	*t *= 0.555	0.579
Gravidity	** **		*χ*^2 ^= 9.654	0.297
0	87 (47.5%)	32 (53.3%)		
1	36 (19.7%)	7 (11.7%)		
2	19 (10.4%)	4 (6.7%)		
3	17 (9.3%)	11 (18.3%)		
4	13 (7.1%)	3 (5.0%)		
5	4 (2.2%)	3 (5.0%)		
6	5 (2.7%)	0 (0%)		
7	1 (0.5%)	0 (0%)		
8	1 (0.5%)	0 (0%)		
Parity	** **		*χ*^2 ^= 0.048	0.826
Nullipara	113 (61.7%)	38 (63.3%)		
Multipara	70 (38.3%)	22 (36.7%)		
Cesarean section	** **		*χ*^2 ^= 0.234	0.629
NO	160 (87.4%)	51 (85.0%)		
YES	23 (12.6%)	9 (15.0%)		
Body mass index BMI (kg/m^2^)	21.7 ± 2.88	21.7 ± 3.82	*t *= 0.093	0.926
Height (cm)	** **			
Mean ± SD	160 ± 4.92	160 ± 5.54	*t *= −0.324	0.746
History of abdominal surgery	** **		*χ*^2 ^= 0.199	0.655
NO	151 (82.5%)	51 (85.0%)		
YES	32 (17.5%)	9 (15.0%)		
Relapse	** **		*χ*^2 ^= 0.996	*P *> 0.999
NO	180 (98.4%)	60 (100%)		
YES	3 (1.6%)	0 (0%)		
Preoperative hemoglobin (g/l)	124 ± 12.5	125 ± 11.6	*t *= −0.521	0.603
Preoperative CA125	30.0 ± 30.0	24.6 ± 22.8	*t *= 1.278	0.203
Other tumor markers	** **		*χ*^2 ^= 0.004	0.949
Normal	138 (75.4%)	45 (75.0%)		
Abnormal	45 (24.6%)	15 (25.0%)		
Cyst location	** **		*χ*^2 ^= 0.612	*P *= 0.434
Right	90 (49.2%)	33 (55.0%)		
Left	93 (50.8%)	27 (45.0%)		
Largest diameter of ovarian cysts				
Mean ± SD	8.74 ± 3.85	8.12 ± 3.41	*t *= 1.106	*P *= 0.270
Deduced pathological type	** **		*χ*^2 ^= 13.782	*P *= 0.019
Mature cystic teratoma	87 (47.5%)	37 (61.7%)		
Endometrioma	38 (20.8%)	3 (5.0%)		
Serous cystadenoma	30 (16.4%)	5 (8.3%)		
Mucinous cystadenoma	4 (2.2%)	3 (5.0%)		
Mesosalpinx cyst	1 (0.5%)	0 (0%)		
Parovarian cyst	10 (5.5%)	5 (8.3%)		
Simple cyst	11 (6.0%)	6 (10.0%)		
Others	2 (1.1%)	1 (1.7%)		
Ultrasound findings	** **		*χ*^2 ^= 3.832	*P *= 0.288
Mixed cyst-solid	100 (54.6%)	39 (65.0%)		
Dense spot echo	29 (15.8%)	4 (6.7%)		
Cystic	42 (23.0%)	14 (23.3%)		
Multilocular	12 (6.6%)	3 (5.0%)		

The data are expressed as the mean ± SD or n (%).

### Development and validation of the predictive model

**Figure 2:**
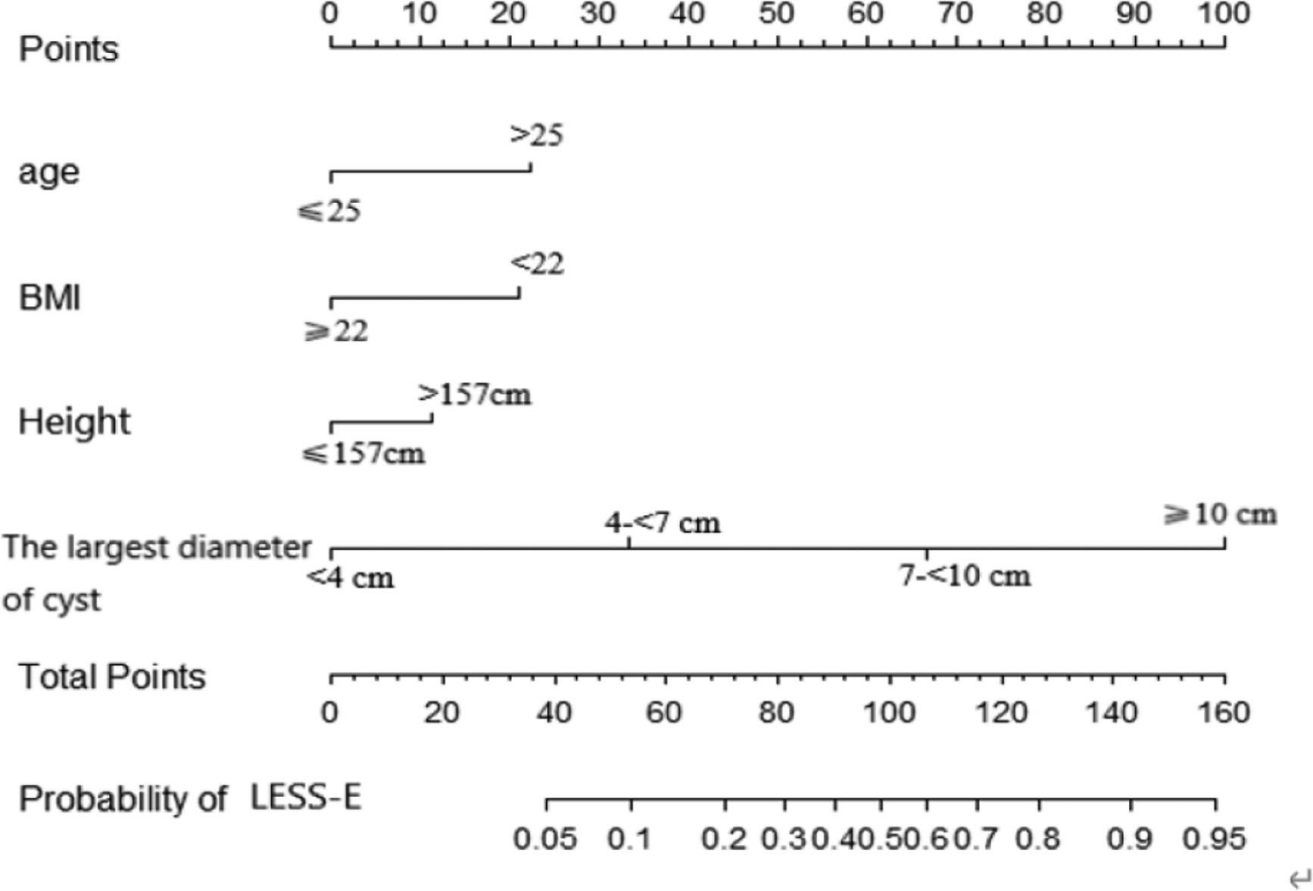
The proposed nomogram to predict the probability of the LESS-E approach. Points were assigned for age, BMI, height and largest diameter of the cyst by drawing a line upward from the corresponding values to the “points” line. The sum of these four points, plotted on the “Total points” line, corresponds to the probability of the LESS-E approach.

**Figure 3:**
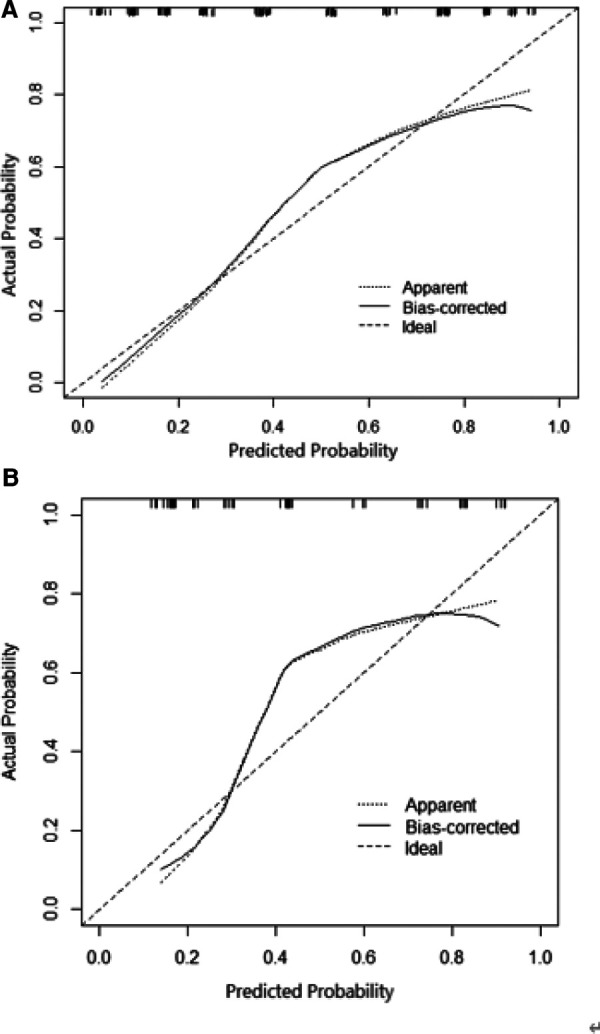
(**A**) Calibration of the nomogram to predict the need for the LESS-E approach for benign ovarian cysts in the training set. (**B**) Calibration of the nomogram to predict the need for the LESS-E approach for benign ovarian cysts in the training set.

**Figure 4:**
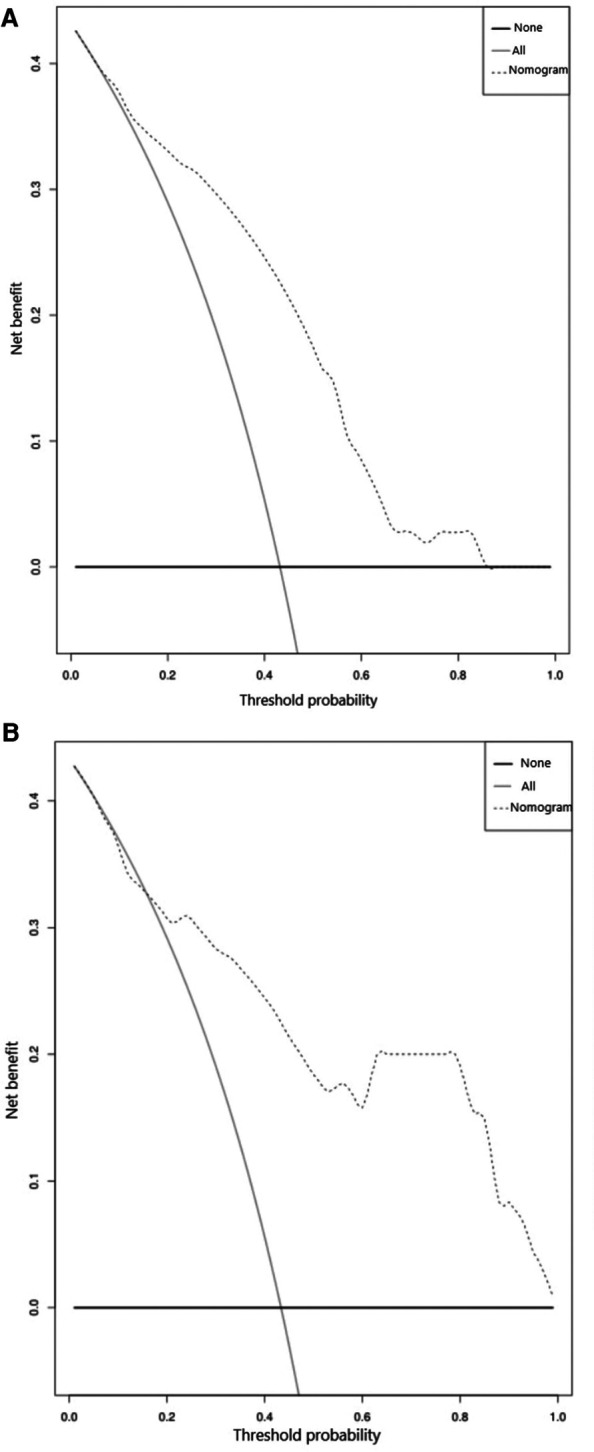
(**A**) Decision curve analysis plot of the nomogram in the training set. The dotted line indicates the clinical net benefits according to the threshold probabilities, the horizontal line assumes that no cases will require the LESS-E approach, and the gray line assumes that all cases will require the LESS-E approach. (**B**) Decision curve analysis plot of the nomogram in the validation set.

In the training set, ROC analysis was performed using age, height, BMI and the largest diameter of the cyst. According to the ROC curve (Supplementary Figure S1), the maximum value of the Youden index was selected as the boundary value. Patients were divided according to age (≤25 years old and > 25 years old), BMI (<22 and ≥22 kg.m^2^), height (≤157 cm and >157 cm), and the largest diameter of the cyst (<4, 4-<7, 7-<10, and ≥10 cm). A multivariate analysis including all preoperative indicators was then performed using logistic regression (Table 2). Based on the results of the multivariate regression analysis and clinical factors, age, BMI, height, and largest diameter of the cyst were incorporated into the multivariable model (Table 3). A nomogram was built to predict the probability of LESS-E cystectomy (Figure 2). The model showed good discrimination (*C*-index, 0.838, *R*^2 ^= 0.415). To use the nomogram, we first drew a vertical line to the point axis according to the value of the rad-score on the corresponding axis. Then, the value of points from other variables was added, and a vertical line was drawn from the total point axis to determine the probability of LESS-E cystectomy at the lowest line. The calibration curve of both the training set and validation set indicated good consistency between the actual and predicted probability of LESS-E cystectomy (Figure 3), which means that the nomogram based on preoperative indicators is a reliable tool for the prediction of LESS-E cystectomy. The DCA plot in the training set showed good positive net benefits in the predictive nomogram model among the majority threshold probabilities (Figure 4) and better positive net benefits in the validation set (Figure 4).

**Table 2:** Multiple logistic regression analysis.

Variable	*β*	*S.E.*	Wald	*P*	Exp (*β*)
Age	1.013	0.525	3.726	0.054	2.753
Gravity	−0.052	0.188	0.076	0.782	0.949
Parity	0.316	0.719	0.193	0.660	1.372
Cesarean section	1.229	1.144	1.154	0.283	3.418
BMI	−1.230	0.450	7.482	0.006	0.292
Height	−0.493	0.453	1.187	0.276	0.611
**Abdominal surgery**	−0.976	1.012	0.929	0.335	0.377
**Deduced pathological type** ^a^			6.265	0.509	
** Mature cystic teratoma**	−0.769	2.145	0.128	0.720	0.464
** Endometrioma**	−0.384	2.202	0.030	0.861	0.681
** Serous cystadenoma**	0.387	2.173	0.032	0.859	1.472
** Mucinous Cystadenoma**	−0.864	2.457	0.124	0.725	0.421
** Mesosalpinx cyst**	19.963	40,192.970	0.000	1.000	467,744,449.105
** Parovarian cyst**	−1.827	2.385	0.587	0.444	0.161
** Simple cyst**	−1.023	2.296	0.198	0.656	0.360
**Preoperative hemoglobin (g/l)**	0.003	0.017	0.043	0.835	1.003
**CA125**	−0.006	0.008	0.519	0.471	0.994
**Other tumor markers**	0.339	0.496	0.468	0.494	1.403
**Cyst location**	−0.247	0.404	0.375	0.540	0.781
**Largest diameter of the cyst (cm)**	1.779	0.308	33.316	0.000	5.925
**Ultrasound findings** ^b^			2.628	0.453	
** Mixed cyst-solid**	−0.134	1.020	0.017	0.896	0.875
** Dense spot echo**	0.338	1.004	0.113	0.737	1.402
** Cystic**	−0.865	0.900	0.923	0.337	0.421
**Constant**	−2.713	3.101	0.765	0.382	0.066

^a^ Taking “other” as a reference.

^b^ Taking “multilocular” as a reference.

**Table 3:** Predictive factors of the LESS-E approach in multivariate analysis.

	*β*	*S.E.*	Wald	*P*	Exp (*β*)
Age	1.098	0.403	7.412	0.006	2.998
BMI	−1.030	0.380	7.335	0.007	0.357
Height	−0.556	0.409	1.850	0.174	0.573
Largest diameter of the cyst	1.642	0.263	38.882	0.000	5.163
Constant	−3.262	0.732	19.876	0.000	0.038

### Establishment of a predicting scoring system (PSS)

**Figure 5:**
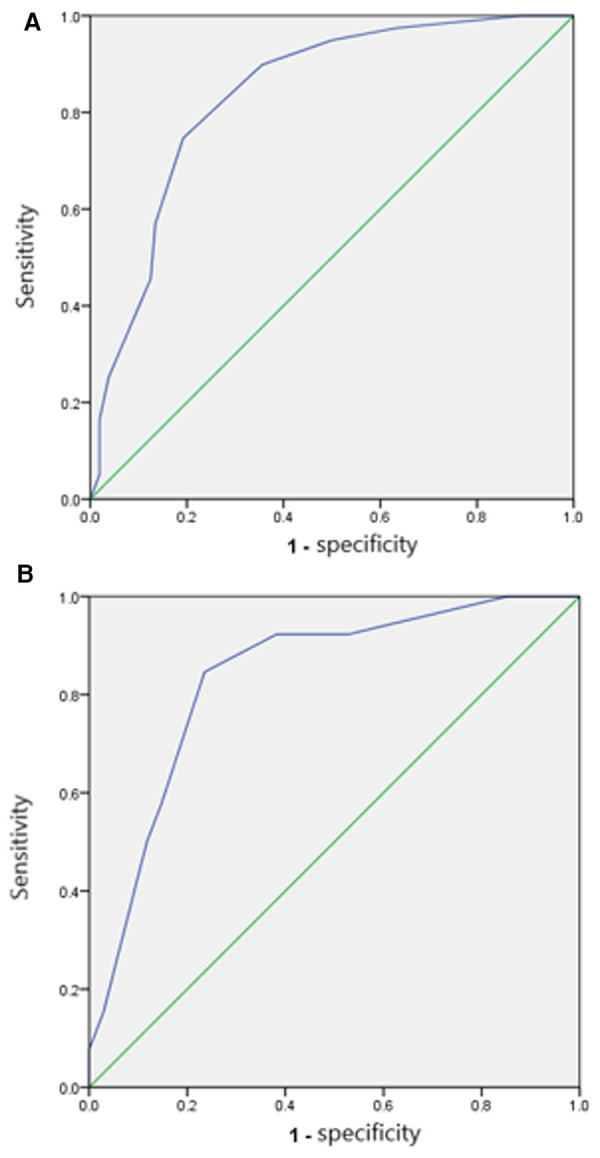
(**A**) ROC curve of the model in the training set. The AUC was 0.837 (95% CI: 0.779–0.895). (**B**) ROC curve of the model in the validation set. The AUC was 0.835 (95% CI: 0.730–0.940).

To simplify the score, the predicted indicators in the regression model were scored by dividing the beta coefficient by the absolute value of the minimum beta coefficient (Table 3). The predictive score derived from each preoperative indicator is shown in Table 4. Patients over 25 years old got 2 points, while others got 0 point; Two points was given to patients whose BMI was less than 22, otherwise 0 point. Patients shorter than 157 cm are given 1 point, otherwise 0 point. Patients with the largest diameter of ovarian cysts ≥ 10 cm were given the highest score (9 points), those with diameters 7-<10 cm were given 6 points, those with diameters 4-<7 cm were given 3 points, and those with diameters <4 cm were given 0 points. The total score of each predictor was used to establish the PSS, which ranged from 0 to 14 points. Receiver operating characteristic (ROC) curve analysis of both the training set and validation set was used to assess the ability of the PSS to predict the probability of LESS-E cystectomy. The AUC related to the PSS was 0.837 (95% CI: 0.779∼0.895) in the training set and 0.835 (95% CI: 0.730∼0.940) in the validation set (Figure 5).

**Table 4:** Final score of predictive index parameters, including 4 features.

Predictive index parameter	standard	Point value
Age	≤25	0
	>25	2
BMI	<22	2
	≥22	0
Height	≤157 cm	1
	>157 cm	0
Largest diameter of the cyst	<4 cm	0
	4-<7 cm	3
	7-<10 cm	6
	≥10 cm	9

### Optimal threshold of the PSS

According to the ROC curve of the total set, the specificity, sensitivity, negative predictive value, and positive predictive value of the PSS under different score thresholds are shown in Table 5. The selected cutoff value according to the maximum point of the Youden index was 8, with a positive predictive value of 67.4% and a negative predictive value of 88.6%, and a preoperative score ≥ 8 identified patients undergoing LESS-E cystectomy.

**Table 5:** Predictive index model.

PIV	Sensitivity	Specificity	Youden index	PPV (%)		NPV (%)	
≥3	100	1.4	0.014	43.6	105/241	100	2/2
≥4	100	9.4	0.094	45.7	105/230	100	13/13
≥5	100	11.6	0.116	46.3	105/227	100	16/16
≥6	96.2	39.1	0.353	54.6	101/185	93.1	54/58
≥7	94.3	52.9	0.472	60.4	99/164	92.4	73/79
≥8	88.6	67.4	0.560	67.4	93/138	88.6	93/105
≥9	70.5	81.9	0.524	74.7	74/99	78.5	113/144
≥10	55.2	87.0	0.422	76.3	58/76	71.9	120/167
≥11	43.8	88.4	0.322	74.2	46/62	67.4	122/181
≥12	22.9	96.4	0.192	82.8	24/29	62.1	133/214
≥13	16.2	97.8	0.140	85	17/20	60.5	135/223
≥14	5.7	98.6	0.043	75	6/8	57.9	136/235

## Discussion

This is the first study based on clinical characteristics that predicted the probability of LESS-E cystectomy for patients with benign ovarian cysts. According to the multivariate logistic regression analyses and clinical factors, age, BMI, height, and the largest diameter of the cyst were confirmed as individual predictive indices for LESS-E cystectomy. We can infer that patients with a lower BMI, shorter height, and larger ovarian cyst diameter had a closer distance between the ovarian cysts and umbilicus, which is more conducive to exteriorization. A PSS was finally established, and patients with preoperative scoring ≥ 8 were recommended for LESS-E cystectomy.

A previous study (27) showed that 7 patients with a diameter of ovarian cysts > 10 cm successfully underwent laparoendoscopic single-site extracorporeal cystectomy with an average operative time of 76.42 minutes, no overflow of cyst contents, and no perioperative complications. In addition, two retrospective case–control studies (14, 16) compared LESS-E cystectomy with conventional laparoscopy and laparotomy and indicated that LESS-E cystectomy resulted in a shorter operation time, less blood loss and a lower leakage rate. Furthermore, our previous propensity score matching study (23) compared LESS-E cystectomy and LESS-I cystectomy and revealed the same results: LESS-E cystectomy was associated with a shorter operation time and lower leakage rate. Above all, we consider that the main advantage of LESS cystectomy is LESS-E approach.

In the “Basic Principles and Anatomy for the Laparoscopic Surgeon”, the sagittal views illustrate that as a patient's body mass index increases, the distance from the base of the umbilicus to the peritoneum and to the retroperitoneal structures as well increase (28). When the patient is supine, in the sagittal plane of the ovary and umbilicus, the vertical, horizontal and the straight distance between the navel and ovary form a right triangle. The smaller the BMI and the thinner the abdominal wall is, the shorter the vertical distance is. The shorter the height and the horizontal distance is, then the closer the ovary and the umbilicus are, so that we can exteriorize the ovarian cyst easily.

To date, no studies have investigated which patients are most suitable for LESS-E cystectomy. We developed a nomogram to predict the probability of LESS-E cystectomy in patients with benign ovarian cysts. This method has been widely applied for the evaluation of gynecological diseases. A nomogram (29) with a *C*-index of 0.78 was developed to predict the probability of recurrence in patients with borderline ovarian tumors (BOT) who had undergone primary surgery, which may guide follow-up treatment. The C-index of our predictive model was 0.838, which demonstrated satisfactory discrimination. Decision curve analysis, which uses benefit and harm to measure clinical value, is increasingly used in practical applications due to its superiority in helping doctors make better clinical decisions (30). The DCA of our nomogram, which predicts the chances of laparoendoscopic single-site extracorporeal cystectomy in patients with highly probable benign ovarian cysts, exhibited satisfactory positive net benefits among the majority of threshold probabilities, indicating excellent clinical utility.

There are various surgical procedures available to patients with ovarian cysts who are indicated for surgery, such as laparotomy, conventional laparoscopy, ultrasound-guided aspiration and laparoendoscopic single-site surgery. Each surgical approach has its advantages and disadvantages, and none are suitable for all patients. From our retrospective cohort study, the main advantage of laparoendoscopic single-site cystectomy is that it is an extracorporeal procedure that preserves as much normal ovarian tissue as possible and can reduce the leakage rate (23). To confer the greatest benefit to patients, we developed a preoperative scoring system to guide surgical decision-making in patients with benign ovarian cysts and proved the clinical value of the PSS. Patients with a score ≥ 8 were recommended to undergo LESS-E cystectomy, while for patients with a score < 8, conventional laparoscopy, LESS-I surgery or reduced port approach can be performed.

The strength of this article is that this is the first study based on clinical characteristics that predicted the probability of LESS-E cystectomy for patients with benign ovarian cysts. It should be noted that our study has several limitations. This was a single-center retrospective study, the sample size of the validation set was relatively small, and independent external validation is needed. A multicenter randomized prospective study needs to be designed to validate the PSS and adjust it if necessary.

In conclusion, the PSS we established may help to guide surgical decisions for patients with benign ovarian cysts and help them derive the most significant benefit.

## Data Availability

The original contributions presented in the study are included in the article/**Supplementary Material**, further inquiries can be directed to the corresponding author/s.
