# Supplementary material for: A preoperative scoring system to predict the probability of laparoendoscopic single-site extracorporeal cystectomy in patients with benign ovarian cysts
Source: Front Surg. 2022 Oct 26;9:991450. doi: 10.3389/fsurg.2022.991450 (PMC9643379; doi:10.3389/fsurg.2022.991450)
Supplement: Supplementary file 1 [file Table1.docx]

Supplementary Table 1

Baseline characteristics. LESS-E vs. LESS-I

|  | LESS-I (N=138) | LESS-E (N=105) | Statistical parameters | Total (N=243) |
| --- | --- | --- | --- | --- |
| Age (years) | 27.8±8.19 | 27.9±7.76 | *t*=-0.173  *P*=0.863 | 27.8±7.99 |
| Gravidity |  |  | χ^2^=8.491  *P*=0.370^*^ |  |
| 0 | 66 (47.8%) | 53 (50.5%) |  | 119 (49.0%) |
| 1 | 29 (21.0%) | 14 (13.3%) |  | 43 (17.7%) |
| 2 | 10 (7.2%) | 13 (12.4%) |  | 23 (9.5%) |
| 3 | 17 (12.3%) | 11 (10.5%) |  | 28 (11.5%) |
| 4 | 10 (7.2%) | 6 (5.7%) |  | 16 (6.6%) |
| 5 | 2 (1.4%) | 5 (4.8%) |  | 7 (2.9%) |
| 6 | 2 (1.4%) | 3 (2.9%) |  | 5 (2.1%) |
| 7 | 1 (0.7%) | 0 (0%) |  | 1 (0.4%) |
| 8 | 1 (0.7%) | 0 (0%) |  | 1 (0.4%) |
| Parity |  |  | χ^2^=0.219  *P*=0.740 |  |
| Nullipara | 84 (60.9%) | 67 (63.8%) |  | 151 (62.1%) |
| Multipara | 54 (39.1%) | 38 (36.2%) |  | 92 (37.9%) |
| Cesarean section |  |  | χ^2^=1.172  *P*=0.279 |  |
| NO | 117 (84.8%) | 94 (89.5%) |  | 211 (86.8%) |
| YES | 21 (15.2%) | 11 (10.5%) |  | 32 (13.2%) |
| BMI |  |  |  |  |
| Mean±SD | 22.4±3.05 | 20.7±3.00 | *t*=4.258  *P*<0.001 | 21.7±3.13 |
| Height (cm) |  |  |  |  |
| Mean ±SD | 160±4.97 | 159±5.19 | *t*=1.154  *P*=0.250 | 160±5.07 |
| History of abdominal surgery |  |  | χ^2^=1.651  *P*=0.199 |  |
| NO | 111 (80.4%) | 91 (86.7%) |  | 202 (83.1%) |
| YES | 27 (19.6%) | 14 (13.3%) |  | 41 (16.9%) |
| relapse |  |  | χ^2^=0.681  *P*=0.580^*^ |  |
| NO | 137 (99.3%) | 103 (98.1%) |  | 240 (98.8%) |
| YES | 1 (0.7%) | 2 (1.9%) |  | 3 (1.2%) |
| Preoperative hemoglobin (g/L) | 124±11.9 | 123±12.8 | *t*=0.575  *P*=0.566 | 124±12.3 |
| CA125 | 25.9±26.5 | 32.3±30.5 | *t*=-1.741  *P*=0.083 | 28.7±28.4 |
| Other tumor markers |  |  | χ^2^=0.388  *P*=0.533 |  |
| Normal | 106 (76.8%) | 77 (73.3%) |  | 183 (75.3%) |
| Abnormal | 32 (23.2%) | 28 (26.7%) |  | 60 (24.7%) |
| Cyst location |  |  | χ^2^=0.546  *P*=0.460 |  |
| Left | 67 (48.6%) | 56 (53.3%) |  | 123 (50.6%) |
| Right | 71 (51.4%) | 49 (46.7%) |  | 120 (49.4%) |
| Largest diameter of the cyst(cm) | 7.24±3.04 | 10.4±3.88 | *t'*=-6.781  *P*<0.001 | 8.58±3.75 |
| deduced pathological type |  |  | χ^2^=13.364  *P*=0.041^*^ |  |
| Mature cystic teratoma | 80 (58.0%) | 44 (41.9%) |  | 124 (51.0%) |
| Endometrioma | 19 (13.8%) | 22 (21.0%) |  | 41 (16.9%) |
| Serous cystadenoma | 13 (9.4%) | 22 (21.0%) |  | 35 (14.4%) |
| Mucinous cystadenoma | 3 (2.2%) | 4 (3.8%) |  | 7 (2.9%) |
| Mesosalpinx cyst | 0 (0%) | 1 (1.0%) |  | 1 (0.4%) |
| Parovarian cyst | 10 (7.2%) | 5 (4.8%) |  | 15 (6.2%) |
| Simple cyst | 11 (8.0%) | 6 (5.7%) |  | 17 (7.0%) |
| Others | 2 (1.4%) | 1 (1.0%) |  | 3 (1.2%) |
| Ultrasound findings |  |  | χ^2^=6.430  *P*=0.092 |  |
| Mixed cyst-solid | 88 (63.8%) | 51 (48.6%) |  | 139 (57.2%) |
| Dense spot echo | 15 (10.9%) | 18 (17.1%) |  | 33 (13.6%) |
| Cystic | 29 (21.0%) | 27 (25.7%) |  | 56 (23.0%) |
| Multilocular | 6 (4.3%) | 9 (8.6%) |  | 15 (6.2%) |
