# Supplementary material for: A preoperative scoring system to predict the probability of laparoendoscopic single-site extracorporeal cystectomy in patients with benign ovarian cysts
Source: Front Surg. 2022 Oct 26;9:991450. doi: 10.3389/fsurg.2022.991450 (PMC9643379; doi:10.3389/fsurg.2022.991450)
Supplement: Supplementary file 2 [file Table2.docx]

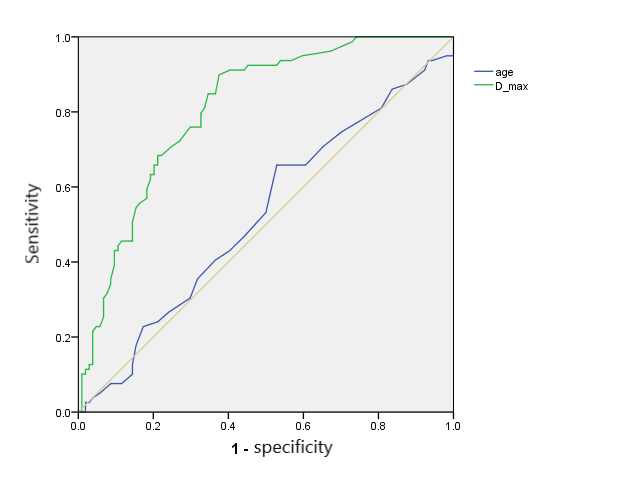


Supplementary Figure 1.1: Receiver operating characteristic (ROC) curve of age and largest diameter of the cyst to predict the surgical type in the training set.


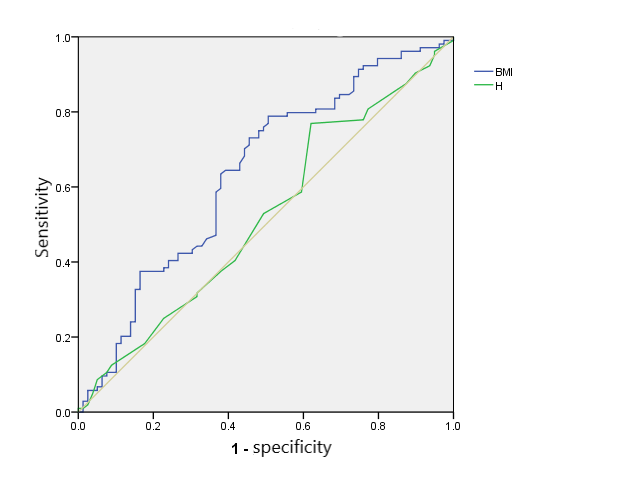


Supplementary Figure 1.2: Receiver operating characteristic (ROC) curve of BMI and height to predict the surgical type in the training set.
